# Gestational diabetes as a risk factor for pancreatic cancer: a prospective cohort study

**DOI:** 10.1186/1741-7015-5-25

**Published:** 2007-08-16

**Authors:** MC Perrin, MB Terry, K Kleinhaus, L Deutsch, R Yanetz, E Tiram, R Calderon, Y Friedlander, O Paltiel, S Harlap

**Affiliations:** 1 Department of Psychiatry, School of Medicine, New York University, 550 1st Avenue, New York, NY 10017, USA; 2 Department of Epidemiology, Mailman School of Public Health, Columbia University, 722 West 168th Street, New York, NY 10032, USA; 3 New York State Psychiatric Institute, 1051 Riverside Avenue, New York, NY 10032, USA; 4 Unit of Epidemiology, The Hebrew University-Hadassah School of Public Health, Ein Kerem, Jerusalem, 91120, Israel

## Abstract

**Background:**

Diabetes is known to be associated with cancer of the pancreas, though there is some debate as to whether it is a cause or a consequence of the disease. We investigated the incidence of pancreatic cancer in a cohort of 37926 Israeli women followed for 28–40 years for whom information on diabetes had been collected at the time they gave birth, in 1964–1976, in Jerusalem. There were 54 cases of pancreatic cancer ascertained from the Israel Cancer Registry during follow-up.

**Methods:**

We used Cox proportional hazards models to adjust for age at baseline and explore effects of other risk factors, including ethnic groups, preeclampsia, birth order and birth weight of offspring.

**Results:**

We observed no cases of pancreatic cancer in the women with insulin dependent diabetes; however, there were five cases in the women with gestational diabetes. The interval between the record of diabetes in pregnancy and the diagnosis of pancreatic cancer ranged from 14–35 years. Women with a history of gestational diabetes showed a relative risk of pancreatic cancer of 7.1 (95% confidence interval, 2.8–18.0).

**Conclusion:**

We conclude that gestational diabetes is strongly related to the risk of cancer of the pancreas in women in this population, and that gestational diabetes can precede cancer diagnosis by many years.

## Background

Cancer of the pancreas is the fourth highest cause of death from cancer among women in the US [1]. It is generally diagnosed at an advanced stage. Only a small proportion of tumors can be surgically resected [2], and many are resistant to chemotherapy or radiation [2,3]. Thus, the lethality of pancreatic cancer is high, with the mortality rate (9.2/100000) approximating the incidence rate (9.5/100000) among women [4]. Diabetes is well known to be associated with pancreatic cancer [5,6]. There has been a long-standing debate, however, as to whether this is a consequence or an antecedent of the pancreatic tumor; evidence exists supporting both views [3]. On the one hand, patients with newly diagnosed pancreatic cancer frequently have type 2 diabetes mellitus of recent onset; removal of the tumor often ameliorates its manifestations [3]. On the other hand, individuals with long-standing diabetes have also been shown to be at increased risk of pancreatic cancer [3].

During pregnancy, women become progressively more insulin resistant as a result of weight gain and release of placental hormones [7]. While most women are able to compensate with increased secretion of insulin and experience only minor changes in plasma glucose levels, those with gestational diabetes mellitus are unable to compensate for the increased resistance and become hyperglycemic [7]. Risk factors for gestational diabetes mellitus include older age, family history of diabetes and high body mass index (BMI) and ethnicity [8]. In the US, the prevalence of gestational diabetes mellitus is approximately 7% [9], though it varies by ethnic group. It is more common among African-Americans, Hispanics, Asians and Native Americans than among non-Hispanic Whites [10-14]. Short-term consequences include fetal macrosomia and other neonatal morbidities; long term sequelae place the mother and offspring at increased risk of type 2 diabetes mellitus [9]. A few investigators have studied gestational diabetes mellitus and gestational glucose intolerance as risk factors for breast cancer and other cancers [15,16] but none, to our knowledge, have investigated gestational diabetes mellitus in relation to pancreatic cancer.

## Methods

This study relies on an ongoing population-based cohort study derived from births, with follow-up till the present day of all offspring and their parents. The Jerusalem Perinatal Study recorded all 92408 births in 1964–1976 to residents of a defined geographic area. Subsets of mothers were interviewed in 1965–1968 (N = 11467 births) in antenatal clinics and in 1974–1976 (N = 16912 births) 1–3 days after birth [17]. The present analysis focuses on the mothers of the 84781 offspring born in the three largest obstetric units, where the study included active surveillance of maternal and obstetric conditions; data on maternal and obstetric information was copied from the labor ward log at the time of birth using separate rubrics in the Perinatal Study's pre-coded forms [17] that allowed for a record of maternal "diabetes" (presumed to be insulin-dependent juvenile diabetes or type 1) and "pre-diabetes", corresponding, approximately, to gestational diabetes mellitus. In that era, all pregnant women were screened for glycosuria at each antenatal visit; if found positive, they would be referred for an oral glucose tolerance test.

In 2004–2005, using the national identity numbers that are assigned to citizens of Israel, we traced and ascertained the vital status of 40898 mothers in this cohort through linkage with Israel's National Population Registry. Then, we linked the cohort to the Israel Cancer Registry. The Israel Cancer Registry, which was started in 1961, is 95.7% complete for pancreatic cancer [18]. Names, identity numbers and other identifying information were removed from the file that was analyzed collaboratively in New York and Israel. The study was approved by the Institutional Review Boards of both participating institutions.

### Statistical analyses

We included all cases of first primary malignant pancreatic cancers, as defined by the International Classification of Diseases for Oncology, 3rd edition (site code: C25 and fifth digit morphology code of 3) that were diagnosed between the first observed birth and 31 December 2004. We used Cox proportional hazards models to estimate the relative risk (hazard ratio) of pancreatic cancer in women with a diagnosis of gestational diabetes mellitus in any pregnancy compared to women without a diagnosis of gestational diabetes mellitus during the entire study period (1964–1976) using the PHREG procedure available in SAS 9.0 (SAS Institute Inc, Cary, NC, USA). Women were followed from the "baseline birth" (i.e., first time they gave birth in 1964–1976) until death, date of diagnosis of any cancer, or end of follow-up period (31 December 2004).

Variables included in the model were those that altered the age-adjusted estimate of the relative risk by more than 10%. We also tested whether a covariate for time period (year of first observed birth) affected the estimate, or was predictive of pancreatic cancer risk. As it did not effect the estimate and did not predict pancreatic cancer risk, we did not include the term in the final model. Age at the first observed birth was treated as a continuous variable, missing values (N = 50) being assigned to the mean (26.2 years). Other variables were treated as dichotomies, or sets with a dummy, coded 1 (if present) or 0 (if absent). Variables tested included categories of birth order at the last observed pregnancy, ethnic ancestry based on the woman's father's place of birth (Israel, other Western Asia, North Africa and Europe etc. – the latter including the Americas, sub-Saharan Africa and Australasia; no information was available for the origin of the woman's mother), social class (based on husband's occupation at last observed birth), categories of education, presence of other specific complications of pregnancy in any observed birth, and birth defects, low (< 2.5 kg) or high (4.0 kg or more) birth weight in one or more offspring. Unless otherwise stated, categories of missing data in other variables (most affected less than 0.1% of women) were included in the reference groups. The results are presented as relative risks, adjusted for age or for other variables, with 95% confidence intervals.

### Numbers and exclusions

Of the 40898 women traced, 37980 (92.9%) delivered at least once in one of the three largest hospitals in Jerusalem, where complete diabetes information was collected for the cohort. Untraced women were similar in age at first observed birth, more often unmarried and more likely to be of European ancestry. Untraced women had a lower prevalence of gestational diabetes mellitus (0.4%) compared to a prevalence of 1.0% among women who were traced. Among women who were successfully traced, the incidence of pancreatic cancer was not significantly related to hospital of birth. We also excluded 41 women who were diagnosed with various malignancies prior to their first observed birth in the study, and an additional 13 women (none with pancreas cancer) who were diagnosed with gestational diabetes mellitus in one pregnancy and type 1 diabetes mellitus in another pregnancy during the study period.

## Results

The assumption of proportionality over time was met based on a visual inspection of log negative log survival plot. There were 37926 women available for study, including 410 diagnosed with gestational diabetes during one or more pregnancies in 1964–1976. Table 1 compares the characteristics of women with and without this pregnancy complication. Women with gestational diabetes tended to be older at the baseline birth, multiparous at the last birth and of slightly higher social class. While there were subtle differences in ethnic ancestry (women with gestational diabetes mellitus being more likely to have a father from Europe), immigrant women were similar to those born in Israel. Several complications of pregnancy were significantly more frequent among women with gestational diabetes mellitus (e.g. heart disease and preeclampsia) and sub-optimal outcomes were more common in their offspring, including low birth weight, high birth weight and congenital malformations.

**Table 1:** Percent distribution of women with and without a history of gestational diabetes, by selected variables

**Characteristic**		**Gestational diabetes**		**p-Value**
			
		**-**	**+**	
Number of women		37516	410	
Percent		100	100	
Age at first observed birth	< 25	47.2	32.4	< 0.0001
	25–29	28.0	29.3	
	30–34	14.5	23.4	
	35+	10.3	14.9	
Birth place of woman	Born in Israel	46.8	47.8	0.7
	Born abroad	53.2	52.2	
Birth place of woman's father	Israel	14.6	11.5	0.05
	Other West Asia	28.3	27.6	
	North Africa	21.5	19.5	
	Europe etc.	35.5	41.5	
Social class at last observed birth	Low	30.8	25.6	0.03
	Middle	37.2	42.7	
	High	32.0	31.7	
Heart disease at any observed birth	No	99.3	96.8	< 0.0001
	Yes	0.7	3.2	
Preeclampsia at any observed birth	No	97.2	87.8	< 0.0001
	Yes	2.8	12.2	
Birth order at last observed birth	1	22.1	11.7	< 0.0001
	2–3	44.7	48.5	
	4+	33.2	39.8	
Any offspring with birth weight ≤4000 g	No	88.7	73.7	< 0.0001
	Yes	11.3	26.3	
Any offspring with birth weight < 2500 g	No	89.0	80.0	< 0.0001
	Yes	11.0	20.0	
Any offspring with congenital malformations	No	92.5	88.3	0.001
	Yes	7.5	11.7	

At the cut-off date of 31 December 2004, the mean length of follow-up was 38.0 years and the median age of the women in the cohort was 59 (43–94). During the 28–40 years of follow-up, 54 cases of pancreatic cancer were reported to the Israel Cancer Registry in the cohort. The interval between diagnosis of gestational diabetes mellitus and diagnosis of pancreatic cancer ranged from 14–35 years. The median age at diagnosis of pancreatic cancer was 57 (range 35–77); among those women who were also diagnosed with gestational diabetes mellitus, the median age at diagnosis was 58 (range 42–76). The life-table estimates of 1-year and 5-year survival rates of these cases were 30% and 8%, respectively.

Table 2 shows the association of selected variables with the relative risk of pancreatic cancer, adjusted for age at entry. Immigrants did not differ from Israeli-born women and although there were differences associated with ethnic origin. The lowest incidence of pancreas cancer was seen in women whose fathers were born in North Africa or Israel. Though the middle and lowest social classes, compared to the highest social class, were at increased risk of pancreatic cancer, the estimates did not reach the level of significance.

**Table 2:** Incidence of pancreatic cancer, age-adjusted relative risk (RR) and 95 % confidence interval (CI) by selected characteristics

		**Pancreatic cancer**			
				
**Characteristic**		**-**	**+**	**RR**	**95% CI**
Number of women		37872	54		
Birthplace of woman	Born abroad	20136	33	1	
	Born in Israel	17736	21	1	0.6–1.8
Maternal father's country of birth	Israel	5535	4	0.4	0.1–1.2
	Other West Asia	10720	21	0.9	0.5–1.7
	North Africa	8136	6	0.4	0.2–0.9
	Europe	13481	23	1	
Social class	High	12141	9	1	
	Middle	14099	23	2.1	1.0–4.5
	Low	11632	22	1.7	0.8–3.7
Birth order at last observed birth	1–2	17664	13	1	
	3+	20208	41	1.3	0.7–2.6
Heart disease in any observed birth	No	37598	51	1	
	Yes	274	3	7.3	2.3–23.4
Preeclampsia in any observed birth	No	36774	51	1	
	Yes	1098	3	1.4	0.4–4.7
Offspring ≥ 4000 g at birth in any observed birth	No	33529	43	1	
	Yes	4343	11	1.7	0.9–3.3
Offspring < 2500 g at birth in any observed birth	No	33679	45	1	
	Yes	4193	9	1.7	0.8–3.5
Offspring with congenital malformations in any observed birth	No	35016	48	1	
	Yes	2856	6	1.6	0.7–3.8

There was a sevenfold increase in risk estimated for women reported to have heart disease in at least one of their pregnancies (p < 0.0008), based on three cases. Other complications of pregnancy (i.e., preeclampsia, low birth weight, high birth weight and birth defects and high birth order) were associated with modest (non-significant) increases in the incidence of pancreatic cancer.

Type 1 diabetes was reported at the time of birth in 137 women; none of these were subsequently diagnosed with pancreatic cancer during the follow-up period. Among the 410 women with gestational diabetes, five were diagnosed with pancreatic cancer. Table 3 shows estimates of the relative risk of this malignancy in women previously diagnosed with gestational diabetes mellitus, both age-adjusted and further adjusted for other variables. There was a highly significant eightfold increase in incidence when taking age into account; this excess was maintained sevenfold and was also highly significant after adjusting for other risk factors such as birth of offspring with especially low or high birth weights.

**Table 3:** Numbers of women with and without pancreatic cancer, relative risk (RR) and 95% confidence interval (CI) by gestational diabetes

**Characteristic**	**Pancreatic cancer**		**Age-adjusted RR**	**95% CI**	**p-Value**	RR*	**95% CI**	**p-Value**
							
	**-**	**+**						
Number of women	37872	54						
Gestational diabetes:								
-	37467	49	1			1		
+	405	5	7.9	3.1–19.8	< 0.0001	7.1	2.8–18.0	< 0.0001

* RR adjusted for age at first observed birth and birth weight.

## Discussion

To our knowledge this is the first study to relate pancreatic cancer in women to gestational diabetes mellitus. The results suggest that gestational diabetes mellitus could be an important risk factor for pancreatic cancer. Unlike type 2 diabetes mellitus, gestational diabetes mellitus has been little studied as a risk factor for cancer. In a population-based case control study that examined pregnancy characteristics and breast cancer, it was reported that the risk associated with gestational diabetes mellitus was reduced in the first five years after pregnancy but somewhat increased after five years [15]. A prospective cohort study in Scotland studied indices of sub-clinical glucose intolerance among women recruited during pregnancy, relating them to cancer 20 years later [16]. The risk of any neoplasm increased with higher fasting plasma glucose levels. Similar results were found for breast cancer. Though that study was quite small (N = 753), it suggested that even subclinical gestational glucose intolerance might be an important determinate of cancer risk.

The conversion rate for gestational diabetes mellitus to type 2 diabetes mellitus can be as high as 70% (range 2.6–70%) depending on the length of follow-up [19]. Women who have had gestational diabetes mellitus are often more insulin resistant than their normal counterparts after pregnancy and have also been found to have a defect in β-cell function (reviewed in Buchanan [7]). Increasing insulin resistance coupled with a decrease in β-cell function over time could lead to hyperglycemia characteristic of type 2 diabetes mellitus.

Several studies have investigated the role of glucose levels and risk of pancreatic cancer. A prospective study (follow-up 12 years) in Chicago found that mean post-load plasma glucose levels were lower among women who subsequently died of pancreatic cancer compared to survivors, though there were only six deaths reported [20]. Another study in the same population in Chicago (average follow-up of 25 years), examined post-load plasma glucose levels in 15183 women, relating them to pancreatic cancer mortality [21]. The authors reported a non-significant increase in deaths from this cause associated with post-load plasma glucose levels above 119 mg/dl. In a prospective study among 468615 Korean women enrolled in a health plan and followed for up to 10 years, mortality from pancreatic cancer was associated with a significant increase in risk among women with fasting serum glucose levels above 90 mg/dl (p = 0.01) [22]. The relative risk (compared to < 90 mg/dl) of fasting serum glucose in the normal range (90–109 mg/dl) was 1.5 (95% confidence interval (CI) 1.2–1.8) and up to 2.1 (95% CI 1.4–2.9) for those ≥ 126 mg/dl; the findings were similar when examining the association between fasting serum glucose levels and the incidence of pancreatic cancer. The results of the study in Korea suggest, as did the study in Scotland [16], that even glucose levels in the upper range of normal could be associated with an increased risk of some cancers, including pancreatic cancer.

There is a pathway by which hyperglycemia could increase the risk of pancreatic cancer in persons with either type 1 or type 2 diabetes mellitus. Hyperglycemia increases the generation of reactive oxygen species (ROS) that are hypothesized to be linked to some of the more common morbidities associated with diabetes [23]. Persons with type I and type 2 diabetes mellitus have been reported to generate more reactive oxygen species (ROS) than controls [24]. If anti-oxidant mechanisms are overwhelmed and DNA is damaged, this could lead to the loss of function in critical proteins such as those involved in tumor suppression. However, it should be considered that in our sample only women diagnosed with gestational diabetes mellitus went on to develop pancreatic cancer whereas no cases of pancreatic cancer were found among women diagnosed with type 1 diabetes mellitus.

Gapstur and colleagues have proposed a mechanism by which hyperinsulemia, associated with type 2 diabetes mellitus, might act to increase the risk of pancreatic cancer [21]. They postulate that in the hyperinsulemic state, the exocrine cells of the pancreas are exposed to extremely high levels of insulin. Insulin at high levels binds to the insulin-like growth factor I (IGF1) receptor [25] and downregulates the IGF binding protein 1, (IGFBP1) [26], thus the amount of bioavailable IGF-1 could increase. Insulin has been reported to increase cell growth in pancreatic cancer cell lines [27], while IGF1 has been shown to increase pancreatic cancer cell growth [28].

For many years there has been a controversy whether diabetes is causally associated with pancreatic cancer or is merely a consequence of tumor growth. Different studies have addressed this question by examining the length of time between diagnosis of diabetes and pancreatic cancer. In a recent meta-analysis, time between diagnoses was examined; it was reported that there was a significant 50% increase in risk associated with type 2 diabetes mellitus diagnosed 5 or more years earlier [6]. In the current study the time between diagnosis of gestational diabetes mellitus and that of pancreatic cancer ranged from 14–35 years. As disease progression is aggressive in pancreatic cancer (the 1-year survival rate was 30% in this cohort) it makes it unlikely that gestational diabetes diagnosed 14 or more years earlier is a consequence of tumor growth [6].

An intriguing observation is the strong association of pancreatic cancer with heart disease. Although based on only three cases, it is unlikely to be due to chance. We have no information on the exact diagnosis; however, heart disease recorded at the time of the birth would have been due to congenital defect, rheumatic heart disease or cardiomyopathy.

Our study has the advantage of a prospective design, long follow-up and complete obstetric history on all births. The data on gestational diabetes mellitus and other birth complications were taken from medical records at the time of birth and did not rely on self-report. Further, the ascertainment of pancreatic cancer is likely to be complete and unbiased relative to the obstetric history. Unlike many studies of diabetes, we were able to distinguish between women with type 1 diabetes mellitus and those with gestational diabetes mellitus; however, we do not have an exact definition of diabetes and modern criteria for gestational diabetes mellitus were not applied in that era. Data were available only on observed pregnancies, and there might be some misclassification of exposure, but it was likely random and would only tend to weaken the association. We were unable to control for smoking history and body mass index in the analysis; however, the prevalence of current smoking and obesity in a subgroup of women interviewed postpartum was only 13.2% and 2.1% respectively. Though there were only 54 cases of pancreatic cancer ascertained through the follow-up period, given the strength of the association our results are unlikely to be due to chance or confounding, although it cannot be ruled out as the RR at the low end of the confidence interval was 2.8.

## Conclusion

The prevalence of gestational diabetes mellitus is reported to be increasing [29,30], not surprisingly given the prevalence of overweight and obesity in the US [31]. Other than type 2 diabetes mellitus, the sequelae of gestational diabetes mellitus remains largely unknown. If the results of the current study are confirmed, then it would be expected that the incidence of pancreatic cancer could increase. Research is urgently needed to ascertain this, and other possible health sequelae of gestational diabetes mellitus, so that appropriate interventions can be developed and implemented.

## Competing interests

The author(s) declare that they have no competing interests.

## Authors' contributions

MCP analyzed and interpreted the data and wrote the first draft of the manuscript and critically revised the manuscript. MBT, KK, LD, RY, ET and RC participated in the analysis and interpretation of the data and critically revised the manuscript. YF contributed to the conception and design of the study and acquisition of the data and critically revised the manuscript. OP contributed to the conception and design of the study and critically revised the manuscript. SH acquired the data and conceived of the design of the study, participated in the analysis and interpretation of the data and critically revised the manuscript. All authors gave final approval of the manuscript.

## Pre-publication history

The pre-publication history for this paper can be accessed here:


